# Effectiveness of the Community Nurse Case Manager in Primary Care for Complex, Pluripathological, Chronic, Dependent Patients: A Study Protocol

**DOI:** 10.3390/nursrep15060191

**Published:** 2025-05-29

**Authors:** Virginia Iglesias-Sierra, Natalia Sánchez-Aguadero, José Ignacio Recio-Rodríguez, Benigna Sánchez-Salgado, Luis Garcia-Ortiz, Rosario Alonso-Domínguez

**Affiliations:** 1 Unidad de Investigación de Atención Primaria de Salamanca (APISAL), 37005 Salamanca, Spain; viglesiass@saludcastillayleon.es (V.I.-S.); donrecio@usal.es (J.I.R.-R.); benissanchez@gmail.com (B.S.-S.); lgarciao@usal.es (L.G.-O.); ralonsod@usal.es (R.A.-D.); 2 Instituto de Investigación Biomédica de Salamanca (IBSAL), 37007 Salamanca, Spain; 3 Gerencia de Atención Primaria de Salamanca, Gerencia Regional de Salud de Castilla y León (SACYL), 37007 Salamanca, Spain; 4 Departamento de Enfermería y Fisioterapia, Universidad de Salamanca, 37007 Salamanca, Spain; 5 Red de Investigación en Cronicidad, Atención Primaria y Promoción de la Salud (RICAPPS), 37005 Salamanca, Spain; 6 Departamento de Ciencias Biomédicas y del Diagnóstico, Universidad de Salamanca, 37007 Salamanca, Spain

**Keywords:** multimorbidity, functional dependence, case management, primary health care, nursing

## Abstract

**Background:** The ageing of the population and the progressive increase in chronic diseases represent a major challenge for healthcare systems. The community nurse case manager (CNCM) is emerging as a key figure to provide comprehensive and continued care for complex and pluripathological chronic patients (CPCPs), especially after hospital discharge. **Objective:** The aim of this study is to pilot CNCMs in assisting CPCPs and assess their effects on functional capacity, cognitive performance, quality of life, readmissions, clinical parameters, satisfaction with home care, and caregiver overload. **Methods:** A comparative study will be carried out at two health centres in Salamanca (Spain). In both centres, CPCPs will continue to receive the interventions included in the Castilla y León Health System Portfolio from their primary care (PC) nurses. In the intervention centre, case management provided by a CNCM will be added. We will recruit 212 CPCPs with cardiac or respiratory disease and/or diabetes mellitus who are dependent for basic activities of daily living and have a programmed hospital discharge. An initial assessment will be performed at home after discharge, followed by assessments at 3, 6, and 12 months. **Expected results:** The intervention is anticipated to improve all study outcomes. **Discussion:** CNCMs may contribute to more proactive and individualised follow-up care for CPCPs and their caregivers, improving care coordination. **Conclusions:** This study will help to evaluate the feasibility and clinical relevance of incorporating the CNCM’s role into PC. This study was registered at ClinicalTrials.gov with the identifier NCT06155591. The date of trial registration was 24 November 2023.

## 1. Introduction

Population ageing is a growing challenge [1,2]. Spain is the fourth European country with the highest proportion of people aged 65 and over, currently accounting for 17% of the total population, with projections reaching 37% in 2049 [3,4]. This trend will lead to an increased prevalence of chronic diseases and, consequently, a higher healthcare demand and resource consumption [5].

Chronic diseases, characterised by their prolonged course (lasting more than six months), slow progression, and multifactorial origin, significantly affect patients’ quality of life [6,7]. Among the most prevalent conditions are cardiovascular and respiratory diseases, as well as diabetes mellitus (DM), which account for 80% of healthcare demand and 90% of mortality in Europe [8]. Furthermore, they represent the leading cause of disability and healthcare expenditure, posing a challenge for both individuals and healthcare systems [9,10].

Complex and pluripathological chronic patients (CPCPs) often present with multiple comorbidities, frailty, and clinical vulnerability, alongside a progressive decline in functional capacity [11,12,13]. This has a significant impact on healthcare demand, with considerable social and economic repercussions [9,14]. The CRONICOM project [15] revealed that 61% of patients hospitalised in internal medicine units were classified as complex chronic patients, and 40% were pluripathological, showing a deterioration in quality of life and an increased dependency following each hospital admission [16,17]. In this regard, various studies highlight the importance of collaboration and coordination between healthcare levels, as well as of the continuity of care, to prevent prolonged hospital stays, early readmissions, and delays in identifying decompensations [18,19,20].

Managing CPCPs requires a comprehensive, personalised, and holistic approach, with continuous monitoring to mitigate the impact of coexisting diseases. The key lies in a multidisciplinary approach that integrates medical treatment, patient education, and social and psychological support [21]. Internationally, several models have been developed for chronic disease care [22], prioritising the effective management of complex chronic cases [23,24,25]. In Spain, regional health services have implemented various strategies and plans for chronic disease care, following the guidelines of the Ministry of Health and scientific societies [26]. Many of these models have incorporated the figure of the nurse case manager (NCM), although its impact has not yet been systematically evaluated [27,28].

Within this role, two areas of practice can be distinguished: the community NCM (CNCM), responsible for managing highly complex patients, with planning and coordination functions mainly within the primary care (PC) setting [29,30], and the hospital NCM (HNCM), who ensures the continuity of care and oversees resource management across different healthcare levels [31].

Although studies on case management in this patient population remain limited, their findings are consistent with improved health-related quality of life, therapeutic adherence, functional capacity, and a more efficient use of healthcare resources, including fewer avoidable hospitalisations and urgency visits [32,33,34,35,36]. Several studies conducted in Spain have also obtained favourable results with this model [37,38]. Moreover, a positive impact on caregiver overload has been reported in some studies through health education, emotional support, and better coordination of care [39,40]. All these effects appear to be greater when implemented in PC [41].

Based on the above, population ageing and the increasing burden of chronic diseases represent a significant challenge that requires solutions in the coming years. The NCM role could play a crucial part in addressing this issue. However, the available studies are limited by short follow-up periods and methodological heterogeneity due to variability in the indicators used and the patient profiles included. Therefore, further research with greater methodological rigour and longer follow-up periods is required.

## 2. Materials and Methods

### 2.1. Objective

The aim of the present study is to pilot CNCMs in assisting CPCPs dependent for activities of daily living (ADLs) with cardiac and/or respiratory pathologies and/or diabetes mellitus from PC and assess their effect on functional capacity, cognitive performance, quality of life, readmissions, clinical parameters, satisfaction with home care, and the overload of the main caregiver.

### 2.2. Design and Setting

A comparative trial will be carried out in the health area of Salamanca (Spain), particularly in the Garrido Sur and Miguel Armijo health centres. Both are urban and have a population with similar characteristics, so they will serve as the intervention and control centre, respectively. The PC nurses at both centres will continue to perform the usual interventions for CPCPs included in the Portfolio of Services of the Castilla y León Health System (SACYL), and case management will be added through a CNCM at the intervention centre.

### 2.3. Participants

This study will include CPCPs who, after being hospitalised due to decompensation of their underlying pathologies, present home care needs at discharge. To be identified as CPCPs, they must meet the criteria established by the Spanish National Health System, which include the presence of two or more clinical categories of pluripathology and at least one complexity criterion. The detailed list of criteria is provided in Table S1.

#### 2.3.1. Inclusion Criteria

The inclusion criteria are CPCPs with associated cardiac and/or respiratory pathologies and/or diabetes mellitus who present frailty criteria (Frail ≥ 1 point), require a main caregiver to perform ADLs (Barthel ≤ 60 points and/or grade II or III dependency recognised by Social Services), require home care and/or social resource management, and agree to sign (themselves or their legal guardians) informed consent to participate in this study.

#### 2.3.2. Exclusion Criteria

The exclusion criteria are patients with other pathologies associated with complex pluripathology, those with non-habitual caregivers, and those who reside outside the area corresponding to the Garrido Sur and Miguel Armijo health centres, despite being assigned to them.

### 2.4. Sample Size Calculation

The sample size was calculated taking into account the main study variables. Specifically, it was estimated to detect a difference equal to or greater than 15 points in the Barthel Index, accepting an alpha risk of 0.05 and a beta risk of 0.2 in a bilateral contrast. Furthermore, the common standard deviation was assumed to be 34.8, as shown in the study by García-Fernández et al. [40], and a loss to follow-up rate of 20% was assumed due to the characteristics of the study subjects and the duration of follow-up. Therefore, it will be necessary to include 212 CPCPs (106 in each group) to detect statistically significant differences.

### 2.5. Variables and Measurement Instruments

Most of the variables will be collected by means of hetero-administered questionnaires, except for those on caregiver overload and user satisfaction, which will be self-administered.

#### 2.5.1. Sociodemographic Variables

Data on age, sex, marital status, type of main caregiver (spouse, children, unpaid caregiver, or paid caregiver), and degree of dependency (II or III) will be collected at the initial assessment.

#### 2.5.2. Primary Outcome Measures

##### Activities of Daily Living (ADLs)

These will be assessed using the Barthel Index, validated in the Spanish population and showing good internal consistency (Cronbach’s alpha = 0.86–0.92), which evaluates a person’s ability to perform ten activities of daily living (eating, dressing and undressing, grooming, using the bathroom, sphincter control, bathing, moving around, and going up and down stairs), assigning up to 15 points to each according to the degree of autonomy. The minimum score is 0 and the maximum is 100. A person is considered totally dependent if their score is ˂20 points; severely dependent if it is between 25 and 60 points; moderately dependent if it is between 65 and 90 points; and mildly dependent if it is equal to 95 points [42].

##### Quality of Life

This will be assessed using the Spanish version of the COOP-WONCA test, which shows acceptable internal consistency (Cronbach’s alpha = 0.73). This instrument relates the patient’s quality of life to their health during the last four weeks by asking about their physical fitness, feelings, daily activities, social activities, state of health, changes in their state of health, and pain by means of a drawing representing a degree of functioning with a 5-level Likert scale. Higher scores express worse levels of functioning/well-being [43].

##### Cognitive Performance

This will be explored through the Montreal Cognitive Assessment (MoCA), which consists of 30 items and lasts approximately 10–12 min. It helps to identify mild impairments in cognitive skills (orientation, short-term memory/delayed recall, executive function/visuospatial ability, language, abstraction, identification, and attention). The total score is 30 points; a score of 26 or higher is considered normal. Cronbach’s alpha for the Spanish version was 0.77 [44].

#### 2.5.3. Secondary Outcome Measures

##### Clinical Variables

To assess some of the clinical variables, the devices listed below will be used and calibrated regularly according to the manufacturer’s recommendations:

- Blood pressure: the measurement will be taken using an OMRON M6 Comfort HEM-7321-E digital blood pressure monitor, taking three readings on the dominant arm with an interval of 1 min between them and using the average of the last two values according to the protocol of the European Society of Hypertension (ESH) [45].
- Heart rate and oxygen saturation: it will be determined using an approved Beurer PO 45 portable pulse oximeter.
- Capillary glycosylated haemoglobin: the test will be performed with a validated Abbott Afinion analyser after obtaining a capillary blood sample by finger prick, using a sampling device that is inserted into the test cartridge [46].
- Capillary blood glucose: a capillary fingerstick blood sample will be taken at least 2 h after the last meal. The test will be performed with an approved Freestyle Glucometer.
- Degree of dyspnoea: this will be examined using the modified Medical Research Council Scale (mMRC), which consists of 5 levels. The higher the level, the lower the tolerance to activity due to dyspnoea [47].
- Symptoms attributable to heart disease: these will be assessed using the New York Heart Association (NYHA) Functional Classification. Class I patients have no symptoms, while those in classes II, III and IV have mild, moderate, and severe symptoms, respectively [48].

##### Number of Hospital Admissions

This will be quantified at all follow-up time points.

##### Number of Drugs Chronically Prescribed

This will be recorded from the electronic prescription of the CPCPs at discharge and at the end of follow-up.

##### Therapeutic Adherence

This will be calculated using a scale designed to assess the patient’s skills and knowledge of the prescribed treatment, with items adapted from the DRUGS and Med-Take scales. For each drug, the following criteria are scored with 1 or 0 points: whether the patient correctly identifies it and knows its indication, dosage, and method of administration. The maximum score is 4, multiplied by the total number of medicines. The assessment of overall adherence is made with the sum of the score obtained for each medication divided by the maximum score [49,50,51]. The scale has not been psychometrically validated in the literature, but it was developed and standardised through expert consensus for institutional use within the regional care programme for polymedicated patients.

##### Primary Caregiver Overload

This will be assessed with the Zarit scale, which consists of 22 questions with answers from 1 to 5 points, with a score ≥ 47 points being considered overburden. High internal consistency was obtained for this questionnaire (Cronbach’s alpha = 0.92) [52].

##### Frailty

The Frail questionnaire, consisting of 5 simple questions on fatigue, endurance, ambulation, comorbidity, and weight loss, will be used. Patients scoring 1 point or more are considered frail. The inter-rater correlation for this instrument was 0.82, indicating good external consistency [53].

##### User Satisfaction

This will be measured through the Satisfad Questionnaire 14, an instrument for evaluation by the patient or main caregiver of the home care services provided, which demonstrated good internal consistency (Cronbach’s alpha = 0.85). Each item is scored on a Likert scale, comprising 4 levels ordered categorically from 0 to 3 (where 0 corresponds to the minimum and 3 to the maximum). The resulting range of possible scores is from 0 to 42 points [54].

### 2.6. Data Collection

Data collection will be carried out by independent evaluators, who will be health professionals from the Salamanca Primary Care Management, with training in the use of the data collection form and the administration of the different tests, which will be paper-based.

The baseline assessment will take place at the patient’s home, and subsequent follow-up assessments will be arranged there or at the health centre depending on their progress:

- Initial assessment: the variables shown in Table 1 will be collected.
- Three-, six-, and twelve-month assessment: the same variables as in the initial assessment will be collected, adding the number of readmissions and medicines prescribed, as well as the evaluation of the satisfaction of the user and/or main caregiver.

### 2.7. Intervention

#### 2.7.1. Common to Both Centres

After planning a hospital discharge, the hospital liaison nurse (HLN) will schedule the CPCP in the agenda of the PC nurse corresponding to the patient in both centres, as usual. All of them will have access to the hospital discharge report via the unified hospital and primary care digital medical record and will contact the patient’s main caregiver to assess their existing needs at home. Moreover, they will continue to perform the usual interventions included in the SACYL Portfolio of Services for CPCP (Figure S1).

Likewise, the evaluators will call the family unit upon discharge from hospital, check the inclusion criteria for this study, explain the research project, and request the signature of the informed consent form if they wish to participate. Subsequently, the initial home assessment will be planned by collecting the data described in Table 1 and the next follow-up assessments at 3, 6, and 12 months will be reported.

#### 2.7.2. Specific to the Intervention Centre

**Figure 1:**
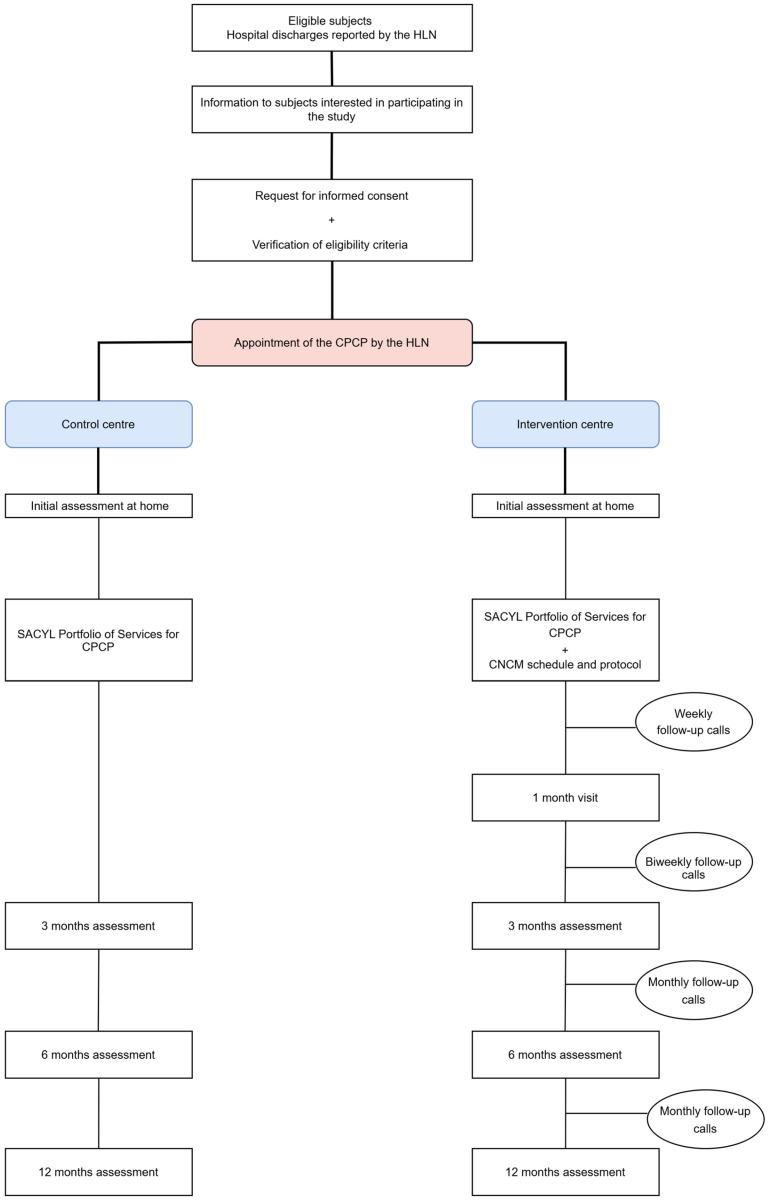
Flowchart of participants’ progress. CPCP: complex and pluripathological chronic patient. CNCM: community nurse case manager. HLN: hospital liaison nurse. SACYL: Castilla y León Health System.

A CNCM action protocol (Figure 1) has been designed to be sequenced according to the circumstances in which the CPCPs find themselves:

- Pre-hospital discharge: The HLN will contact the CNCM to inform them of the imminent hospital discharge.
- Hospital discharge: The HLN will schedule the CPCP on the CNCM’s agenda, and the CNCM will arrange a home visit and carry out a comprehensive nursing assessment based on Marjory Gordon’s functional patterns. The main caregiver will be identified, and health education will be provided to improve home care. An infographic to identify the signs and symptoms of decompensation or exacerbation, as well as a direct-dial phone number, will also be given. The CNCM will then inform the PC team (physician, nurse, and social worker) about the patient’s situation and needs, managing necessary appointments or resources and coordinating care among professionals and levels.
- Post-hospital discharge proactive telephone monitoring: The CNCM will make comfort calls every week during the 1st month, every 15 days until 3 months from recruitment, and every month until 6 and 12 months (Table S2).
- One-month visit: The CNCM will perform a physical examination and assess adherence to treatment, the presence of decompensation/exacerbation of the process, the Barthel Index, and the satisfaction of the user or main caregiver.
- Occurrence of decompensation/exacerbation: If the patients and/or main caregivers report suspicious signs and/or symptoms through the direct dial telephone number, an appointment will be made with their PC physician that same day or, in the event of seriousness, urgency and/or emergency resources will be activated.
- Hospital readmission: The CNCM will be kept informed through the HLN and CPCP’s digital medical record. Once discharged, the process will return to the “hospital discharge” phase, and a new home visit will be scheduled to reassess the current situation.

### 2.8. Statistical Analysis

The normality of the variables will be analysed using the Kolmogorov–Smirnov test. Continuous variables will be presented as mean ± standard deviation, or as median and interquartile range in the case of a non-normal distribution. Qualitative variables will be expressed as number and percentage. The association between qualitative variables will be analysed with the chi-square test or Fisher’s test, as appropriate. Comparisons between quantitative variables will be carried out using Student’s *t*-test or the non-parametric Mann–Whitney U test for independent measurements and paired Student’s *t*-test for related measurements. A repeated-measures analysis of variance (ANOVA) will be performed to assess the effect of the intervention between study centres. Covariates will be included in the model to control for potential confounders such as baseline functional status, comorbidities, or characteristics of the main caregiver. An alpha risk of 0.05 will be set as the limit of statistical significance for bilateral hypothesis testing. Data will be analysed with SPSS statistical software version 28.0.

### 2.9. Strengths and Methodological Limitations of This Study

Although this study follows all the recommendations for non-randomised interventional studies included in the TREND statement, there is a possibility of confounding factors not considered. As this study will be conducted in CPCPs, the loss rate is expected to be high, because their characteristics may prevent them from completing the follow-up due to institutionalisation or death. Given the nature of this study, subjects will not be blinded to the intervention, but the investigator in charge of analysing the data will not be aware of the centre allocation.

## 3. Expected Results

Based on the study objectives and the existing literature, we anticipate that patients in the intervention group will show improvements in functional capacity, health-related quality of life, cognitive performance, clinical parameters, and satisfaction with the care received. In addition, a reduction in hospital readmissions and caregiver burden is hypothesised.

The findings will also provide insight into the feasibility and potential impact of this professional profile in PC, with results interpreted in light of the clinical and social complexity of the target population.

## 4. Discussion

Despite the implementation of different management models and care strategies for patients with multiple chronic conditions, they continue to experience a significant decline in functional capacity, a deterioration in quality of life, delayed detection of signs or symptoms of decompensation, and worsening therapeutic adherence, leading to an increase in hospital readmissions.

Therefore, it is essential to establish health policies that promote comprehensive care based on a collaborative and multidisciplinary approach. These strategies should ensure patient- and family-centred care, fostering shared decision-making. Likewise, it is crucial to actively involve patients in the various processes related to their chronic pathologies, with a particular emphasis on self-care and empowerment. In this context, the NCM emerges as a key figure to lead this model, while PC represents the optimal setting for its implementation, as it is the level of care closest to the community and best positioned to respond to patients’ needs.

In this regard, the incorporation of CNCMs in PC could enhance comprehensive patient and family care. Through home-based monitoring, these professionals would facilitate the early detection of changing needs, favour a prompt response to emerging health issues, and optimise the use of available resources, guaranteeing more coordinated and efficient care. However, despite its potential, experience with this professional role in PC remains limited in Spain. This study aims to generate comparative data that may help assess its applicability in the community context and support decision-making in chronic care planning.

Thus, the recognition of the CNCM as a professional category within the Castilla y León health system, following the results of this project, could contribute to cost reduction and improved quality of care by reducing care fragmentation and ensuring integrated, patient- and environment-centred care. Even so, this study has some limitations, including the non-randomised design and the reduced sample size. Nevertheless, conducting it under real-world conditions will allow for the generation of contextualised evidence.

## 5. Conclusions

This study might help to determine whether interventions led by CNCMs in PC offer benefits compared to usual follow-up for patients with complex chronic conditions and functional dependence. The resulting evidence may support decisions on the organisation of community-based care, promoting models that enhance care continuity, self-care, and health system efficiency.

## Figures and Tables

**Table 1:** Diagram of the study schedule. √ denotes the time points at which each procedure will be carried out. SACYL: Castilla y León Health System.

	Study Period				
	Enrolment		Follow-Up		
Time Point	Preliminary Telephone Contact	Initial Assessment	3 Months Assessment	6 Months Assessment	12 Months Assessment
**Recruitment:**					
Eligibility screen	√				
Informed consent	√	√			
**Intervention:**					
Control condition Usual care according to SACYL portfolio	√	√	√	√	√
Intervention Usual care according to SACYL portfolio + Case management		√	√	√	√
**Data Collection:**					
Sociodemographic variables		√			
Clinical variables		√	√	√	√
Hospital admissions		√	√	√	√
Drugs prescribed		√	√	√	√
Therapeutic adherence			√	√	√
Activities of daily living		√	√	√	√
Quality of life		√	√	√	√
Cognitive performance		√	√	√	√
Caregiver overload		√	√	√	√
Frailty		√	√	√	√
Signs and symptoms of decompensations/exacerbations		√	√	√	√
User satisfaction		√	√	√	√

## Data Availability

The datasets that will be used and/or analysed during the present study will be available upon request from the corresponding author.

## Supplementary Materials

The following supporting information can be downloaded at: https://www.mdpi.com/article/10.3390/nursrep15060191/s1, Figure S1: Interventions for complex pluripathological chronic patients (CPCPs) in the Portfolio of Services of the Castilla y León Health System (SACYL); Table S1: Spanish National Health System criteria to identify complex and pluripathological chronic patients (CPCPs); Table S2: Guidance for proactive telephone follow-up by the community nurse case manager (CNCM).

## Public Involvement Statement

There is no public involvement in any aspect of this research.

## Guidelines and Standards Statement

This manuscript was drafted against the TREND statement for non-randomised interventional studies.

## Abbreviations

The following abbreviations are used in this manuscript:

ADLs	Activities of Daily Living
ANOVA	Analysis of Variance
CNCM	Community Nurse Case Manager
CPCP	Complex and Pluripathological Chronic Patients
DM	Diabetes Mellitus
ESH	European Society of Hypertension
HLN	Hospital Liaison Nurse
HNCM	Hospital Nurse Case Manager
mMRC	Medical Research Council Scale
MoCA	Montreal Cognitive Assessment
NYHA	New York Heart Association
NCM	Nurse Case Manager
PC	Primary Care
SACYL	Castilla y León Health System
