# Supplementary material for: Effectiveness of the Community Nurse Case Manager in Primary Care for Complex, Pluripathological, Chronic, Dependent Patients: A Study Protocol
Source: Nurs Rep. 2025 May 29;15(6):191. doi: 10.3390/nursrep15060191 (PMC12196078; doi:10.3390/nursrep15060191)
Supplement: Supplementary file 1 [file nursrep-15-00191-s001.zip › nursrep-3554243-supplementary.pdf]

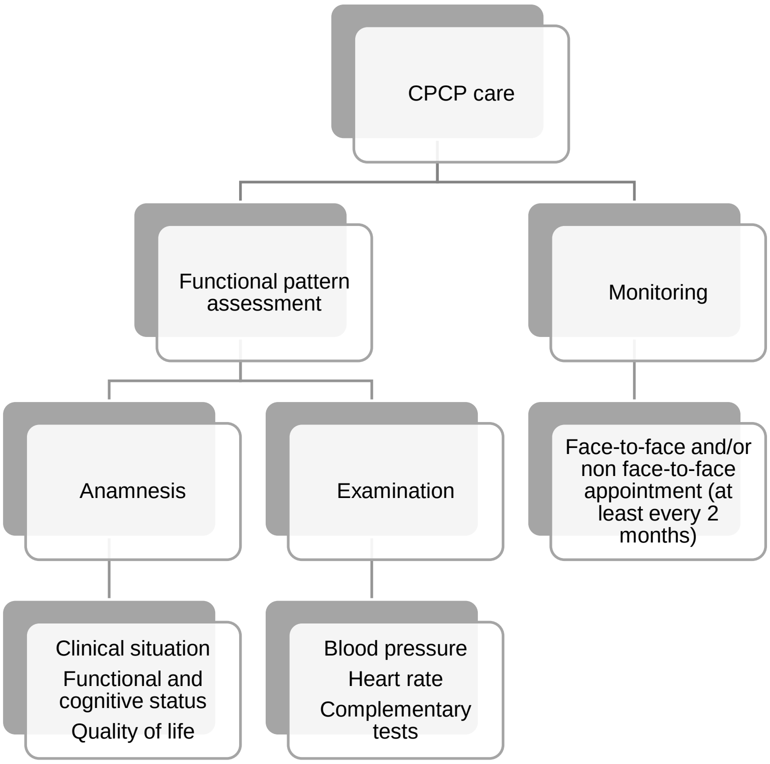

Figure S1. Interventions for Complex Pluripathological Chronic Patient (CPCP) in the Portfolio of Services of the Castilla y León Health System (SACYL). Available in: https://www.saludcastillayleon.es/institucion/es/catalogo-prestaciones/cartera-servicios/cartera-servicios-atencion-primaria.

Table S1: Spanish National Health System criteria to identify complex and pluripathological chronic patients (CPCP).

**PLURIPATHOLOGY CRITERIA** (at least two different must be met)	
**Category**	**Conditions**
A	Heart failure (NYHA II-IV) or ischemic heart disease.
B	Systemic vasculitis or autoimmune disease; chronic kidney disease (GFR <60 ml/min or albumin-creatinine ratio >30 mg/g for ≥3 months).
C	Chronic respiratory disease with MRC grade 2 dyspnoea, FEV1 <65%, or SpO2 ≤ 90%.
D	Chronic inflammatory bowel disease; chronic liver disease with hepatic insufficiency or portal hypertension.
E	Stroke or permanent motor impairment with Barthel <60; or cognitive impairment (MMSE <23).
F	Symptomatic peripheral arterial disease; diabetes with proliferative retinopathy or symptomatic neuropathy.
G	Chronic anaemia (Hb <10 mg/dL twice over >3 months); active neoplasm not eligible for curative treatment.
H	Chronic osteoarticular disease causing limitation in basic ADLs (Barthel <60).
**COMPLEXITY CRITERIA** (at least one must be met)	
• Severity within the listed clinical categories.<br>• ≥1 hospital admission in the last year.<br>• ≥1 fall in the last year.<br>• Malnutrition (BMI <18.5).<br>• Extreme polypharmacy (≥10 chronic prescription drugs).<br>• Barthel index <60 or level II/III dependency without adequate caregiver or with a vulnerable caregiver (≥80 years, with illness/disability, with recognized dependency, or responsible for multiple dependents).	

ADLs: Activities of Daily Living; BMI: Body Mass Index; FEV1: Forced Expiratory Volume in 1 second; GFR: Glomerular Filtration Rate; Hb: Haemoglobin; MMSE: Mini-Mental State Examination; MRC: Medical Research Council; NYHA: New York Heart Association; SpO2: Peripheral oxygen saturation.

Table S2: Guidance for proactive telephone follow-up of the Community Nurse Case Manager (CNCM).

Main Questions	Supplementary Questions
How do you feel?	
Have your main caregiver changed?	
During this time, have you had any of the signs and/or symptoms of decompensation shown in the infographic provided?	It will be briefly explained where they can consult the signs and/or symptoms of their pathology in the infographic.
In relation to the medicines prescribed at discharge, have you had any problem?	If so, what kind of problem have you had, and can I help you in any way?
Did you understand all the information about your illness given to you by healthcare professionals at the time of your discharge from hospital?	
Do you know the care to improve your state of health that was proposed to you at the time of your discharge from hospital?	
Can you carry out the care necessary to perform basic activities of daily living?	
Are you concerned about any issues at this moment?	If so, what is it and how can I help you?
Do you have any hospital consultation scheduled?	If so:<br>• How many appointments do you have with hospital professionals?<br>• Are they face-to-face or telephone appointments?<br>• Which specialist has given you an appointment?<br>• Do you need any analysis or diagnostic test?
